# Supplementary material for: Comparative transcriptomic analysis reveals potential mechanisms for high tolerance to submergence in arbor willows
Source: PeerJ. 2022 Feb 3;10:e12881. doi: 10.7717/peerj.12881 (PMC8818271; doi:10.7717/peerj.12881)
Supplement: Supplemental Information 2 [file peerj-10-12881-s002.zip › Legend for supplemental Figures.docx]

Figure S1 Two physiological indicators of the arbor willow germplasm under submergence stress

a, MDA content of 13 varieties under control and submergence stress; b, total chlorophyll content of 13 varieties under control and submergence stress.

Figure S2 Clustering dendrograms of genes and module detecting

Gene clustering on TOM-based dissimilarity; module division by dynamic tree cut; different colors represent different modules; M module division based on merging similar modules;

Figure S3 Heat map of module-trait relationship

Each row corresponds to a module, and each column corresponds to a trait; the correlation coefficient and the corresponding p-value are shown in the matrix; ME: module eigengenes

Figure S4 Expression heatmap of genes from red and green modules

Figure S5 GO Enrichment pathway of red and green modules
