# Supplementary figures and images for: Comparative transcriptomic analysis reveals potential mechanisms for high tolerance to submergence in arbor willows
Source: PeerJ. 2022 Feb 3;10:e12881. doi: 10.7717/peerj.12881 (PMC8818271; doi:10.7717/peerj.12881)

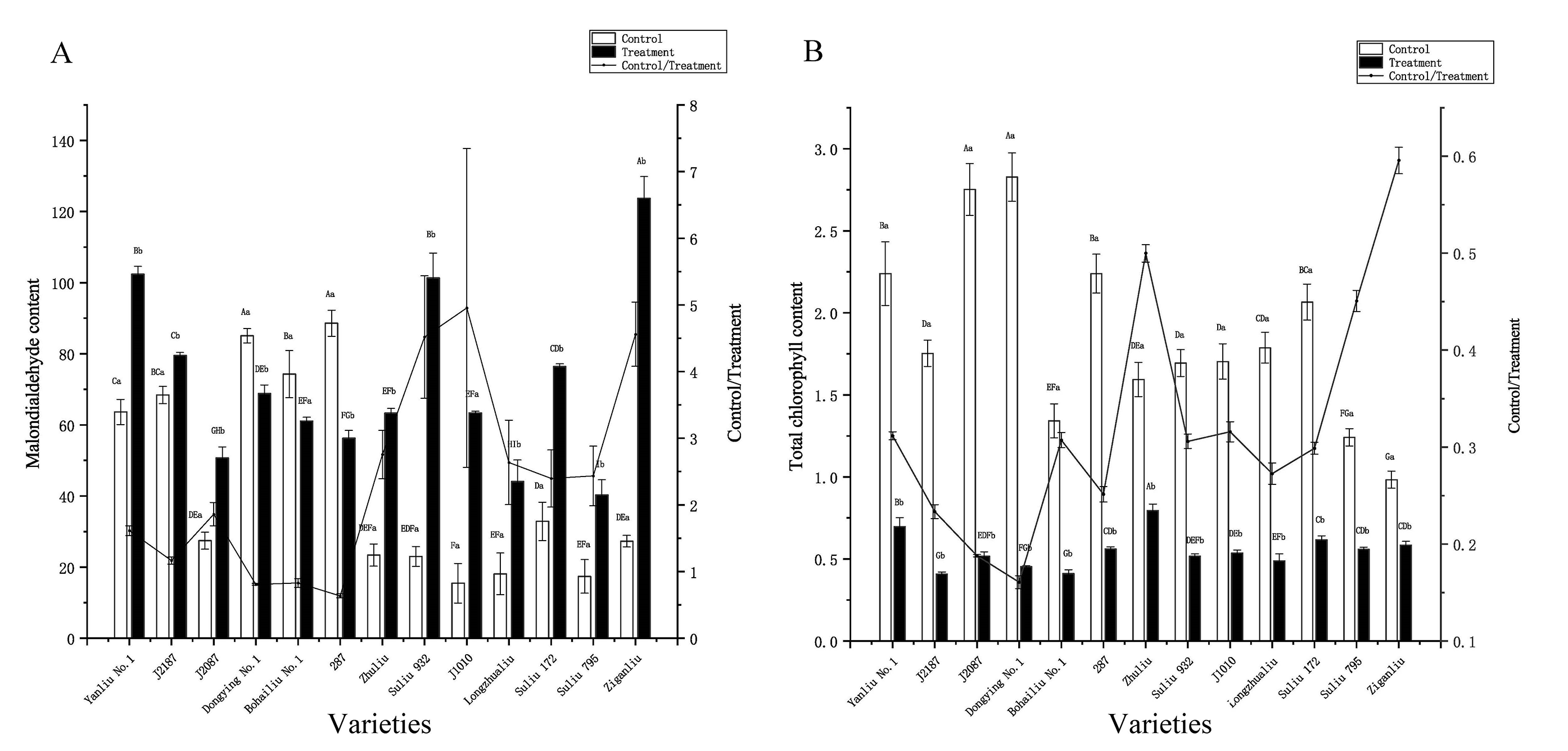

Supplement: Supplemental Information 2 [file peerj-10-12881-s002.zip › Fig S1 Two physiological indicators of the arbor willow germplasm under submergence stress.png]

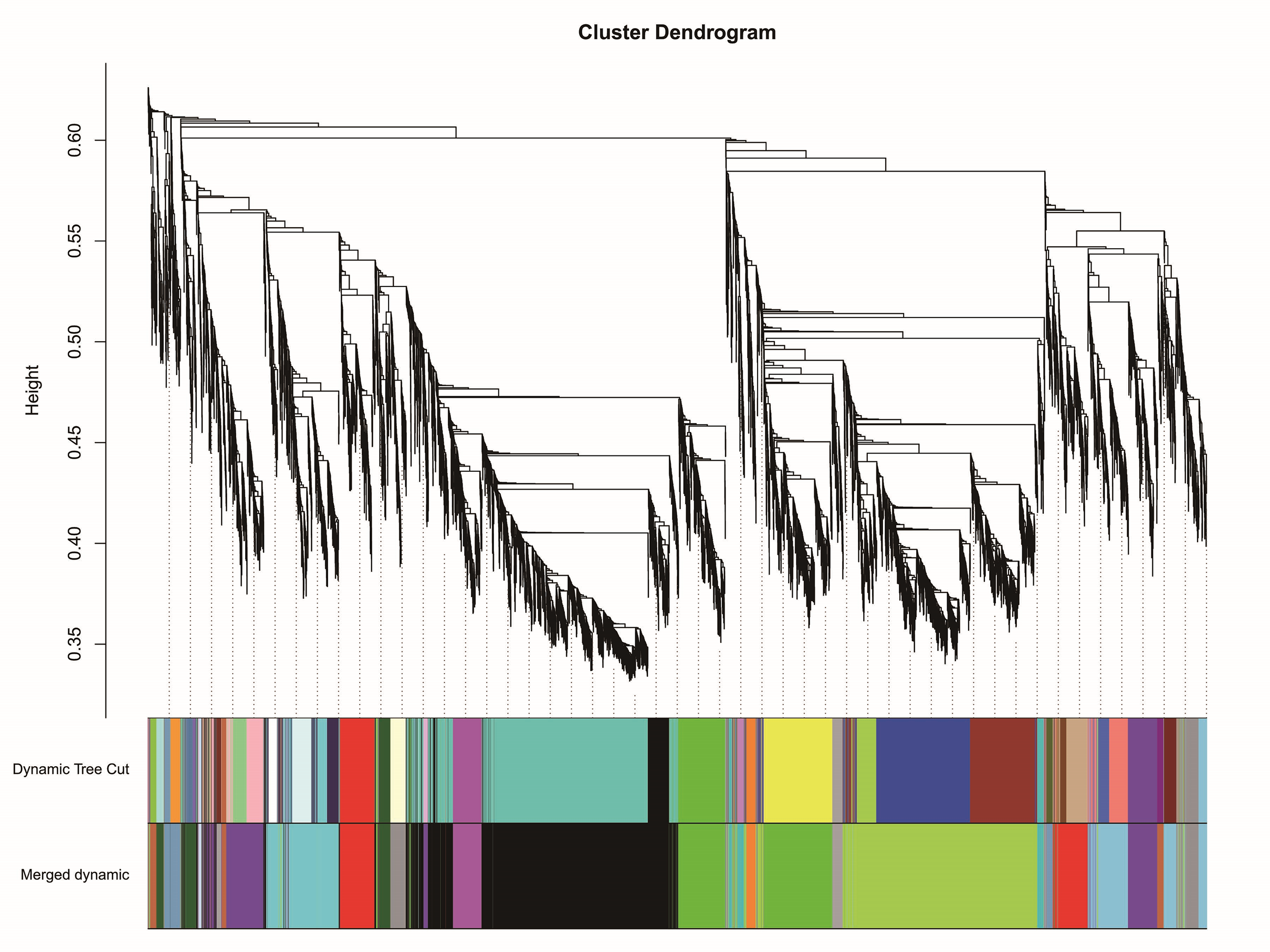

Supplement: Supplemental Information 2 [file peerj-10-12881-s002.zip › Fig S2 mergedModuleTree.png]

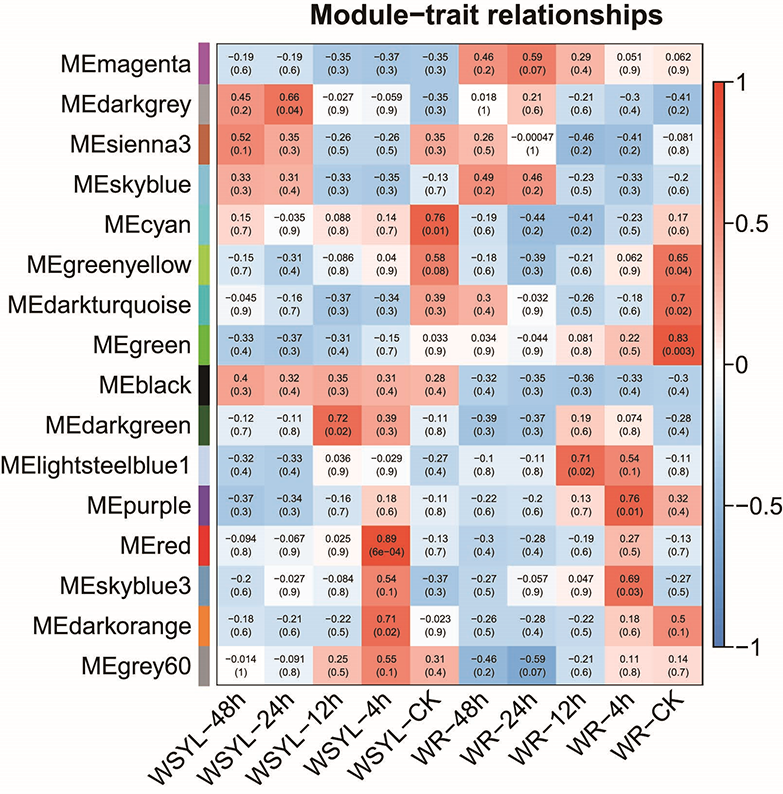

Supplement: Supplemental Information 2 [file peerj-10-12881-s002.zip › Fig S3 Module-Trait_Correlation sample name.png]

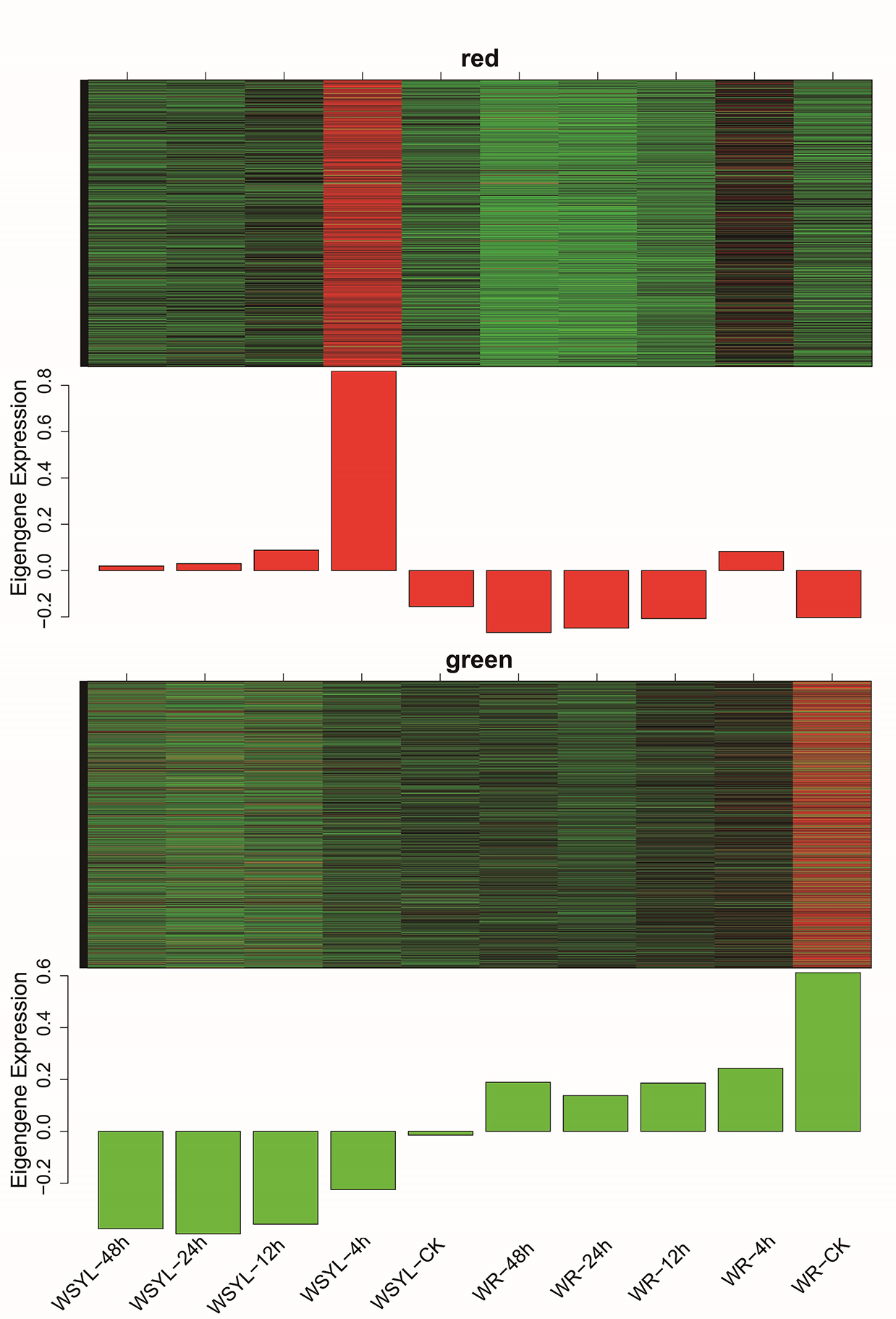

Supplement: Supplemental Information 2 [file peerj-10-12881-s002.zip › Fig S4 GREEN RED MODULES EXPRESSION HEATMAP.png]

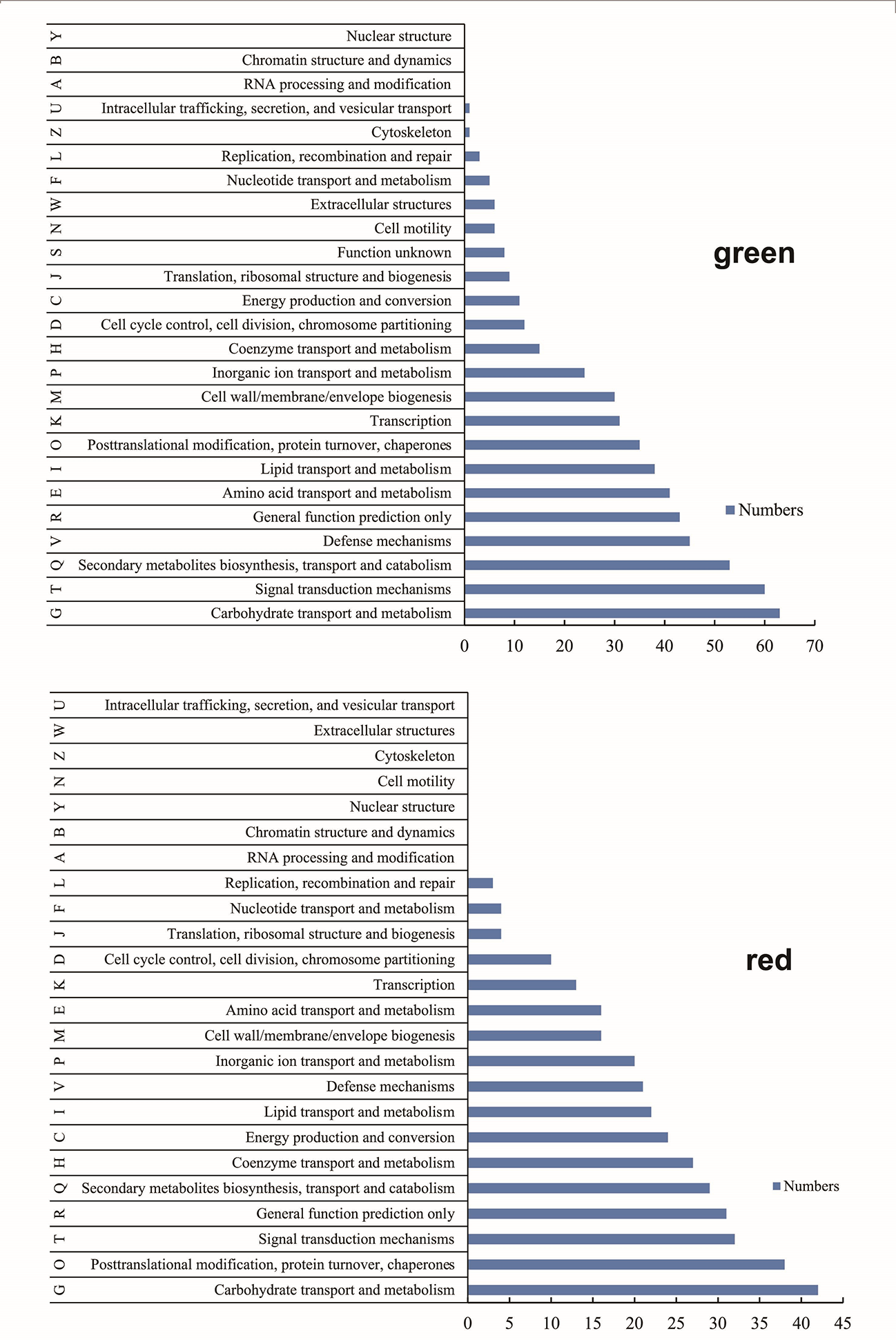

Supplement: Supplemental Information 2 [file peerj-10-12881-s002.zip › Fig S5 GREEN RED MODULES Terms Enrichment.png]
