# Supplementary material for: Comparative transcriptomic analysis reveals potential mechanisms for high tolerance to submergence in arbor willows
Source: PeerJ. 2022 Feb 3;10:e12881. doi: 10.7717/peerj.12881 (PMC8818271; doi:10.7717/peerj.12881)
Supplement: Supplemental Information 3 [file peerj-10-12881-s003.zip › Table S1 qRT-PCR 15 Primers list.docx]

**Table S1 qRT-PCR Primers list**

| Gene name | Annotation | Primer name | Primer Sequence |
| --- | --- | --- | --- |
| EVM0056498 | NAC | EVM0056498-F  EVM0056498-R | CGGAGCGGATAAACCCATTG  TGCCAGCAGACCTATCAACA |
| EVM0054186 | GIF | EVM0054186-F  EVM0054186-R | TCTCAAGTTGCTATGCCCCA  TGGGTGAAGTGTTGCTGTTG |
| EVM0004070 | Trihelix | EVM0004070-F  EVM0004070-R | GAAGCTTTAGGCACCATGGC  ATGGGCTTATTGCGTTGCAT |
| EVM0046362 | Trihelix | EVM0046362-F  EVM0046362-R | GAGGGACATCTTCACCACCA  TGATCATGTGATGCTGCTGC |
| EVM0046399 | AtADH1 | EVM0046399-F  EVM0046399-R | TACTTCTGGGAAGCCAAGGG  TTCAACGATCCCTCCTGCTT |
| EVM0029609 | ACO | EVM0029609-F  EVM0029609-R | ATGGAGAAGCTCAACGGTGA  GGTTCAGCAGCTCGAAGAAG |
| EVM0018193 | AtSUS1 | EVM0018193-F  EVM0018193-R | TACTTCCCCTACACCGAGGA  ATTGGCTTGTTGCGGTCTTT |
| EVM0019131 | CAT1 | EVM0019131-F  EVM0019131-R | CTCTGGGTCAGAAGCTAGCA  TATTCTGCTTGCAATCCGGC |
| EVM0028892 | DLO1 | EVM0028892-F  EVM0028892-R | TTAAAGGTCCTGGCCACGAT  TTACCGATCACTTCCTCCGG |
| EVM0005793 | SARD1 | EVM0005793-F  EVM0005793-R | AGCCGATGATCAGGAGAGTG  TGGTGATGATTCAAGGGCCT |
| EVM0044857 | ICS1 | EVM0044857-F  EVM0044857-R | CTACGGCCACCCTCAATTTG  CTTGTGCTGACAGTGTAGCC |
| EVM0025544 | PCO1 | EVM0025544-F  EVM0025544-R | GGCGCCACCATAAGAAGATG  TCCAGCGCTACAAGAGTCAA |
| EVM0049755 | AP2/ERF | EVM0049755-F  EVM0049755-R | AAGGCATTACCGGGGTGTTA  CGCAGCTTCAATGGCAGTAT |
| EVM0045225 | AP2/ERF | EVM0045225-F  EVM0045225-R | CATGGGGAAAGTATGCTGCC  AACTGCCATCGAACCTCTCA |
| EVM0056506 | WRKY | EVM0056506-F  EVM0056506-R | AGGTGCACTCACAAGTACGA  TGGTGCCCGAGATATGTTGT |
| EVM0015559 (Homolog of SapurV1A.0655s0050.1) | Actin | Sm Actin1-Q-F  Sm Actin1-Q-F | GTCAAGTTCTTTGCTTTCCTCC  CATCACAATCACTCTCCGACTA |
